# The impact of self-directed team on high-performance organization with the mediating role of knowledge sharing culture: Organizational support as a moderator

**DOI:** 10.3389/fpsyg.2022.950204

**Published:** 2022-10-06

**Authors:** Bakr Fakhri Mohammed, Zhao Jingjie, Cong Yang, You Yuwei, Yousra Mahmoud Ali Attia Zalat

**Affiliations:** ^1^Department of Human Resource Management, Jilin University, Changchun, China; ^2^College of Business and Management, Jilin University, Changchun, China

**Keywords:** self-directed team, knowledge sharing culture, organizational support, high-performance organizations, high-tech enterprises

## Abstract

The primary aim of this investigation is to identify the relationship between self-directed teams and high-performance organizations (work). Furthermore, exploring the mediating role of knowledge-sharing culture with the self-directed team and high-performance organization relationships. Moreover, this study analyzed the moderating role of a sense of organizational support in the relationship between knowledge-sharing culture and high-performance organizations. Using the PLS-SEM approach (SmartPLS 3.3.7) in this study, we obtained data from employees of high-tech enterprises listed on the Shanghai Stock Exchange China to analyze this study. The present research seeks to determine the direct and indirect effects of the study-related factors on the outcome of the investigation. According to the findings, a self-directed team positively impacts knowledge sharing culture, knowledge sharing behavior, knowledge sharing strategy, and mutual trust among its members. Knowledge-sharing culture has no mediating role and its component in the relationship between independent and dependent constructs. This study also indicated that a sense of organizational support has no moderating effect on the relationship between a knowledge-sharing culture and high-performance organizations. This study may practically contribute to high-tech enterprises to develop and implement business development and proper self-directed team, knowledge-sharing culture, and sense of organizational support, and consequently, can contribute to the growth of overall high-performance organizations.

## Introduction

The concept of high-performance organizations (HPOs) has received increasing attention in countries all over the globe over the last several decades. China is not an exception to this rule, and the managers of Chinese high-tech enterprises have been seeking factors that would help them elevate their organizations’ performance. On the other hand, there does not seem to be sufficient research on high-performance organizations in Chinese high-tech enterprises. According to a recent review of research comparing high-performance levels in Chinese and Irish firms (de Waal, 2020), only a small number of studies have been conducted in the Chinese setting. The Chinese economy has transitioned from a planned economy to a market socialist state and from an economy that state-owned enterprises primarily ruled to one with a broad range of public and private ownership forms. As a result of these shifts, Chinese businesses are being forced to adjust to remain competitive in the modern Chinese economy (Warner and Rowley, 2013).

When reviewing the relevant research, it was found that studies have been conducted on the self-directed team as a potential predictor of high-performance organizations (Macy et al., 2007). On the other hand, it has been observed that knowledge sharing is a critical aspect in restructuring organizations and helping them to attain higher performance. Knowledge sharing was identified as an essential feature in high-performance organizations by Bhatti et al. (2020), for example. In addition, a culture of knowledge sharing has been employed as a factor that helps to communicate the influence of underlying factors, such as leadership, management practices, and corporate social responsibility, on the performance of a business. Furthermore, Hung et al. (2011) identified a substantial mediating influence of knowledge-sharing culture to improve high-performance organizations.

Studies conducted on high-performance organizations have shown that self-directed teams and knowledge-sharing culture play a vital role in high-performance organizations (Junior and Novaski, 2011; Yiu and Law, 2012). Furthermore, even the literature on the knowledge-sharing culture has demonstrated that self-directed teams have a key influence on knowledge-sharing cultures (Zárraga and Bonache, 2003). However, few studies have investigated the link between self-directed teams, knowledge-sharing culture, and high-performance organizations. This research proposed cultural values to influence high-performance organizations. However, it appears that this transition is missing a connection in the area of knowledge-sharing culture, which has the potential to be a source that helps high-performance organizations nurture the impact of a self-directed team.

This study examines the relationship between a self-directed team, a knowledge-sharing culture, and high-performance organizations. It significantly contributes to managerial and organizational insights into factors influencing high-performance organizations. The basis for this study is the literature that was reviewed, as well as the findings that were presented above. Due to the lack of literature pertinent to the issue, this study aims to analyze the mediating role of knowledge-sharing culture in the relationship between the self-directed team and high-performance organizations. Additionally, this study examines the moderating effect of a sense of organizational support on the relationship between knowledge-sharing culture and high-performance organizations. It is appropriate to investigate this relationship via this study, particularly in a developing market such as China, where there is a lack of previous studies. The researcher believes that a knowledge-sharing culture and high-performance organizations are two of the most important factors that might contribute to the development of the economy of the country (Oyemomi et al., 2019). In addition, the study investigated the role of a knowledge-sharing culture as a mediator between self-directed teams and high-performance organizations. While this research also evaluated the moderating effects of organizational support on the link between knowledge-sharing culture and high-performance organizations. The next sections of the study are organized as follows: the following part includes a literature review and the development of a hypothesis. The methodology, which also covers the data collection technique, is described in the third section, while the conclusions are discussed in the fourth section. The last part discusses the findings with prior research, provides managerial implications, and recommends new avenues of research.

## Literature review and hypothesis development

As the literature examines high-tech enterprises moving toward self-directed teams and knowledge-based economies, knowledge management is becoming increasingly important to the country’s economy. A human-centered approach emphasizes the human components of organizational activity, such as self-directed teams and human resources, and creates an environment that encourages employees to share their expertise. This study discusses the following variables: a self-directed team, knowledge-sharing culture with its dimensions, organizational support, and high-performance organization.

### High-performance organization

The concept of a high-performance organization has been extensively researched. Scholars have, in the past, examined this subject from several angles, with various specific goals and with various interpretations (de Waal, 2021). This reflected the difficulties and the need to provide a unified definition of high-performance organizations (HPO). Since the book by Peters and Waterman, 1982 called “In Search of Excellence,” the notion of a high-performance organization has been widely accepted. They said in their book that high-performance organizations have a strong culture and alignment between strategy, structure, leadership, and workforce skills. Organizations’ competitiveness in the 1990s was typically measured by their ability to adapt to environmental changes and their ability to learn (Schein, 1993).

Understanding the concept of a high-performance organization should be the overall goal of any organization. Similarly, it is very important to fully understand human resource development’s importance and strategic importance in modern business organizations. To compete effectively and sustainably, organizations must constantly adapt to changing conditions by initiating and implementing significant and large-scale changes in how they operate and do business. Otherwise, they risk going out of business (de Waal, 2021). As a result, it is not surprising that businesses are constantly looking for new strategies to produce long-term, high-performance results (de Waal, 2021). Ability to adapt well and react quickly to changes, manage long-term, and incorporate integrated management structure, continuous improvement of core competencies, and value genuine treatment of employees. Assets distinguish a high-performance organization from its peers over an extended period (Do and Mai, 2020).

A significant amount of investigation has been carried out in the field of human resource management and high-performance organizations in China. For example, Liang et al. (2012) and Zhang and Morris (2014) investigated the link between HRM and organizational performance in Chinese-Western joint ventures and owned subsidiaries. They found a positive relationship between firm performance and the degree to which organizations used high performance. Chandra and Chao (2011) also found a positive relationship between HRM practices and performance in Chinese high-tech enterprises. At the same time, Cai et al. (2018) studied Chinese high-tech firms. They concluded that employees’ perceptions of the meaning and importance of their job act as a mediator between servant leaders and innovative work behavior. While Yu et al. (2019) examined the efficiency of high-performance organizations in Chinese companies, the findings showed that high-performing work improved organizational commitment and decreased job withdrawal behaviors and turnover intentions. In addition, high-performance work decreased employees’ likelihood of leaving their jobs.

**FIGURE 1:**
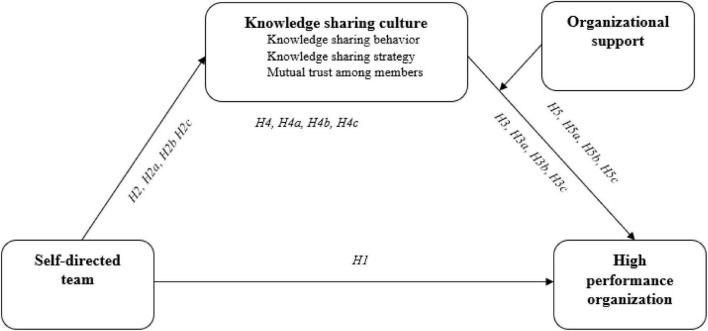
Conceptual framework.

While reviewing the previous studies, it became abundantly clear that the primary focus of those studies was on various aspects of excellence as well as certain methods of improvement. As a result, it was impossible to locate both thorough and statistically verified research in the Chinese context in high-performance enterprises. In high-performance organizations, the link between self-directed teams and knowledge-sharing culture is the subject of study in this particular piece of research. However, this framework (Figure 1) was only evaluated for a small sample of Chinese high-tech enterprises, and no direct relationship was established between the framework and organizational performance. As a result, this framework is not entirely acceptable for use as a comprehensive framework within the Chinese context.

### Self-directed team and high-performance organization

Self-directed work teams are those organizational units in which employees share operationally interdependent tasks and are equally responsible for final products, and individual team members have the wide range of abilities needed to complete tasks that are the collective responsibility of the team and employees who receive input and assessments that are providing in contexts of the team’s performance (Bishop and Scott, 2000). Self-directed teams have a high level of self-determination Taylor et al. (2016), which encompasses control over the speed of work, allocation of duties, work periods, and involvement in hiring and training new team members. Self-directed work teams also have more autonomy than traditional work teams.

A self-directed team is a small number of people, usually workers, responsible for managing their jobs and accomplishing a defined goal (Berezin and Kokernak, 2015). The term ‘self-directed’ in the context of self-directed teamwork can be defined in different ways, some contradictory. In this study, we use the concept of Davis et al. (2004), which is quite broad. Self-directed work teams are defined as “a team that is involved in any decision-making that a manager or supervisor normally does.” Thus, project scheduling, issue resolution, team member selection, and assigning specific tasks to team members are all examples of decisions that can be made. According to Allen et al. (2003), the adoption of self-directed teams is generally seen as a signal that organizations recognize and respect their workers’ contributions. Employees of organizations often perceive this as a sign of the organization’s commitment to them.

Organizations have begun to shift authority from organizations to employees to achieve greater flexibility, reduced response time, and greater operational efficiency (Rigby and Ryan, 2018). This is made possible by reducing organizational hierarchies and creating self-directed teams that are able to respond to specific project needs (Lee and Edmondson, 2017). Especially in high-performance firms, organizational structures have changed from hierarchical and management-based structures to structures emphasizing employee autonomy in contemporary times (Katzenbach and Smith, 2015). These organizations have been called “agile,” “agile,” “low-hierarchy,” and “self-managed,” among other terms (Lemmetty and Collin, 2020). In reality, this means, for example, that these teams are so-called self-directed teams, which are accountable and responsible for the entire project.

When implemented in daily tasks in an organization, self-directed teams motivate employees and increase the organization’s overall productivity (Caruso, 2018). Fapohunda (2013) stated that team building, especially self-directed teams, gives employees more freedom and allows them to participate in decision-making. According to Gilley et al. (2010), organizational teams provide better employee involvement and enhance their goal attainment, ultimately affecting organizational performance. Given the potential impact of a self-directed team on workplace outcomes, deploying such a team involves developing employees’ abilities to use knowledge-based resources, which is useful, as business needs change regularly. It aims to improve the performance of organizations. Geerts et al. (2021) stated that a self-directed team can help increase productivity while improving organizational systems and increasing employee satisfaction. As a result of this discussion, we propose the following hypothesis:

*H1: The self-directed teams positively affect high-performance organizations.*

### Self-directed team and knowledge-sharing culture

The concept of “knowledge sharing culture” is relatively new, even though there is no lack of facts regarding the influence of organizational culture on knowledge management and knowledge sharing. Knowledge culture and knowledge creation culture are two early variations still in use today (Intezari et al., 2017). At the same time, Yi (2019) outlines how organizations can establish and implement the culture and benefits of knowledge sharing. Knowledge-sharing strategies and knowledge behaviors in an organization’s knowledge-sharing culture have all been identified as important components of that culture (Kim and Park, 2020). Dyduch and Krasodomska (2017) stated that the amount of trust between two parties determines the extent to which knowledge is disclosed and how people screen and share. In particular, the independent construct self-directed team and the culture of knowledge sharing, which are taken up in this study, involve distinct elements and are not all related to the same thing, as mentioned earlier. A key difference between them is that a culture of knowledge sharing is concerned with the strategies, procedures, and tools to stimulate the creation of new knowledge and organize content systematically for quick access and efficient use in the organization.

On the other hand, a self-directed team is concerned with rules and procedures for dealing with employees who exhibit desirable behaviors and work for a high-performing organization. Similarly, a culture of knowledge sharing focuses on the shared beliefs and assumptions of all members of the organization’s workforce. While knowledge sharing, behavior, knowledge sharing strategies, and mutual trust are the key characteristics of a knowledge-sharing culture that distinguish it from others.

Self-directed team members must learn many skills to work well independently and with each other. These abilities can be divided into three distinct categories. The first is operational, which is necessary to complete the activities of daily living. The second is social competence for effective communication within teams, also known as interpersonal skills, and the third is improved learning capabilities for innovation and knowledge sharing among teams (Tjepkema, 2003). It favors self-directed and empowered teams that are encouraged to make autonomous decisions and engage in knowledge-sharing and collaborative behavior (Van Wyk, 2017). Members of self-directed work teams appreciate and enjoy the benefit of shared empowerment that builds trust to define the processes required to meet high-performance objectives (Taylor, 2013).

A self-directed team in the workplace is commonly thought of as emerging as a collection of diverse practices whenever employees are involved. Culture is a term that refers to the routines, social conventions, and religious beliefs that underpin everyday activities (Bourdieu, 1990). From a workplace team perspective, culture is a system of activities in which knowledge is not isolated and vice versa (Lemmetty and Collin, 2020). A self-directed team at work can express itself in two ways: independent problem solvers and a source of shared knowledge (Choi and Park, 2014). The skills and abilities of individuals are described using a self-directed team, but the word ‘knowledge’ is used in organizational and employee-level formulations to express knowledge (Lemmetty and Collin, 2020). In organizations, self-directed teams are also viewed as an aggregate of organizational process, task, and goal-oriented elements.

Self-directed work teams consist of organizational employees with a wide range of skills to become more competitive, set up something new, or launch a product launch program. Additionally, these employees are drawn from multiple departments, and team members share their knowledge and experience with their peers to accomplish these goals. During the process of knowledge sharing, each of these teammates acquires knowledge. Additionally, other characteristics define a highly effective self-directed work team, including the following: joint responsibility, interdependence, empowerment, and a common goal are all important concepts. As a result, we propose the following hypothesis:

*H2: The self-directed teams positively affect a knowledge-sharing culture.*

*H2a: The self-directed teams positively affect knowledge-sharing behavior.*

*H2b: The self-directed teams positively affect knowledge-sharing strategy.*

*H2c: The self-directed teams positively affect mutual trust among members.*

### Knowledge sharing culture and high-performance organization

The notion that organizational culture has a positive relationship with organizational performance has long been widely recognized by researchers in the management literature (Imran et al., 2021; Imran and Jingzu, 2022). Many researchers estimate that the knowledge-sharing culture is one of the key factors determining the effectiveness of knowledge management implementation, professionals, and organizations (Intezari et al., 2017). The extent to which collaborative relationships are prevalent in the organizational culture is also an important underlying driver of knowledge sharing (Chen et al., 2014). Collaborative operations from a knowledge-based perspective have recently been researched with the assumption that knowledge sharing is essential to creating innovative techniques, goods, or services, and therefore high-performing organizations in this process performance are enhanced (Hassan and Deen, 2019; Riana et al., 2020).

A culture of knowledge sharing is more enjoyable and productive for organizations. Employees who communicate and share their expertise can meet their work goals and perform their responsibilities more effectively, resulting in higher organizational performance in high-performing organizations (Rehman et al., 2018; Oyemomi et al., 2019). Several previous studies have looked at the effects of knowledge sharing on organizational performance and have reached similar conclusions: knowledge sharing has a significantly positive effect on high-performance work (Atapattu, 2018; Hassan and Deen, 2019). Our research specifically focuses on knowledge-sharing culture, including its dimensions, such as knowledge-sharing behavior, knowledge-sharing strategies, and mutual trust among members. Especially in high-performance organizations where knowledge sharing is widespread, trust and collaborative effort abound and the knowledge-sharing behavior of individuals. As a result, the following hypothesis is presented:

*H3: Knowledge-sharing culture positively affects high-performance organizations.*

*H3a: Knowledge-sharing behavior positively affects high-performance organizations.*

*H3b: Knowledge sharing strategy is positively affecting high-performance organizations.*

*H3c: Mutual trust among members positively affects high-performance organizations.*

### The mediating role of the knowledge-sharing culture

Knowledge is generally recognized as a valuable resource for creating competitive advantages, especially prior to changes in an organization’s operating environment (Omotayo, 2015). Furthermore, knowledge sharing helps improve an organization’s ability to manage knowledge while allowing employees within the business to accomplish a task or goal more quickly and successfully (Le and Lei, 2018). Therefore, knowledge-sharing culture is one of the most important organizational resources that can be used to achieve long-term competitive advantage (Ni et al., 2018). Based on previous research on knowledge sharing, we define knowledge sharing culture as the process of exchanging knowledge and expertise among organizational employees. Which helps individuals equip and complement each other with unique and interesting knowledge and skills to achieve their work and organizational goals. Previous researchers have given different definitions of knowledge-sharing culture (Maizza et al., 2019; Halisa et al., 2021).

Employees’ knowledge-sharing practices are significantly affected by whether they are promoted or discouraged by their self-directed teams. Regarding developing and maintaining a healthy environment of knowledge sharing among workers in a firm, team collaboration is critical (Lee and Kim, 2016). Several studies have indicated that a self-directed team fosters a supportive work environment and also provides appropriate tools for promoting knowledge-sharing activities among employees in a self-directed team environment (Piczak and Hauser, 1996; Vito, 2019). As a result of the positive organizational environment established by a self-directed team, individuals become more innovative and eager to share their unique knowledge capital with their coworkers. According to Jolij’s (2014) findings, a self-directed team places great importance on creating and fostering good employees’ attitudes toward knowledge sharing, collaboration, and fostering a supportive culture.

Many researchers have emphasized the importance of knowledge-sharing culture in improving organizational performance (Ma et al., 2017; Ganguly et al., 2019). To be more specific, Ali et al. (2019) argued that employee knowledge-sharing practices help organizations achieve higher levels of organizational performance. Similarly, Jiang and Liu (2015) presented evidence showing that knowledge sharing among employees is directly associated with high-performance organizations in the workplace. At the same time, high-performance organizations rely heavily on their employees’ knowledge, experience, and skills (Fu et al., 2017). Similarly, they explain that high-performance organizations rely heavily on their employees’ knowledge, skills, and abilities in the value creation process. According to Sánchez et al. (2015), a high-performance organization with strong knowledge-sharing cultures competes more successfully against other organizations in terms of performance. Following the recent findings of Cavaliere et al. (2015), employees can learn and integrate knowledge through a knowledge-sharing culture. As a result, they are better equipped to transfer new ideas into organizational processes due to knowledge sharing. The preceding argument has shown that an organization’s self-directed team and culture of knowledge sharing is an important factor, which in turn has a beneficial effect on the characteristics of a high-performance organization. In light of the above discussion, we propose the following hypotheses:

*H4: Knowledge-sharing culture mediates between the self-directed team and a high-performance organization.*

*H4a: Knowledge-sharing behavior mediates between the self-directed team and a high-performance organization.*

*H4b: Knowledge sharing strategy mediates between the self-directed team and high-performance organization.*

*H4c: Members’ mutual trust mediates between the self-directed team and a high-performance organization.*

### The moderating role of organizational support

According to organizational support theory, the degree to which employees believe their organization appreciates their productivity and concerns about their well-being is called organizational support (Eisenberger et al., 2016). A sense of responsibility to care for the welfare of the organization and help achieve its goals can be achieved by gaining organizational support (Abu Hashish, 2017). Further, organizational support strengthens employees’ beliefs by incorporating employees’ psychological needs, their organizational membership and role status into social identities, which will improve financial and non-financial compensation performance from their employers and increase job satisfaction (Onyeka and Onuoha, 2021). If employees are committed, and their employers desire loyalty to their jobs, assume that organizations provide high levels of support to their employees by mutual cooperation. In this case, employees are more likely to be psychologically committed to their organizations, resulting in less likelihood of turnover and higher job performance (Madden et al., 2015).

Previous empirical research has shown several cases of relationships between organizational support and organizational performance. According to the study conducted by Kawai and Strange (2014), organizational support favors organizational performance. At the same time, Kurtessis et al. (2017) determined that organizational support has the potential to improve overall organizational performance. In addition, several previous studies have reported contradictory results. According to Chen et al. (2020), organizational support was positively associated with better employee performance. The question now is whether organizational support is directly associated with high-performance organizations or if it is moderated by the relationship between knowledge-sharing culture and high-performance organizations. This research aims to examine the moderating role of organizational support in developing high-performance organizations with special emphasis on Chinese high-tech enterprises.

The availability of organizational support as an asset to the organization allows employees to develop positive feelings based on the support and understanding of their coworkers and superiors and to have confidence in their abilities (Wen et al., 2019). Pleasant feelings of this nature relieve employees of emotional fatigue from emotional work. According to researchers, when employees are engaged in emotional labor, organizational support acts as a valuable external energy supply that supports their emotional recovery (Karatepe, 2015). For high-performance organizations to acquire many of the resources necessary to deliver their emotionally labor-based services, organizational support also helps increase their workforce’s confidence and ability to serve customers. Several studies have shown that organizational support can significantly increase employee performance and significantly reduce the absorption and spread of negative thoughts among employees, as well as the true thoughts, and feelings of employees, and reduces the gap between desired positive emotions in customer service (Nixon et al., 2011). It was found by Gabriel et al. (2015) that organizational employees who use deep acting and surface acting strategies use more emotional resources than those who do not use deep acting strategies or surface acting strategies. So, it is more likely that it will increase the happiness and satisfaction of employees if their resources are rewarded or given an extra incentive. Alternative outcomes result in resource mismatches among employees, leading to emotional exhaustion, work stress, and other undesirable outcomes.

When organizational support is perceived as poor, newly promoted employees accumulate negative emotions. According to emotional events theory, employees who experience the dual attack of loss of positive psychological resources and organizational support are more likely to leave their current employers and seek employment with other better organizations. Likewise, employees who feel organizational support are more likely to be cared for, respected, and recognized by their business. The high-level demands of the new generation of employees are addressed to a certain extent, resulting in a generational transfer of pleasant feelings. Because positive emotions promote a culture of knowledge sharing among workers, employees’ physical and mental well-being are more likely to meet the needs of the current situation and reduce the likelihood of new-generation employees leaving the workforce. Is. Wang (2017) conducted an empirical study in which he used perceived organizational support as a moderating variable and found that it had a moderating effect on the effects of psychological capital on workers’ attitudes and actions. In several research studies, organizational support has previously been used as a moderator along with other factors (Mahon et al., 2014; Malik and Noreen, 2015). We argued that the sense of organizational support could act as a moderator between a knowledge-sharing culture with its dimensions and a high-performance organization in this context. As a result, we propose the following hypothesis:

*H5: Organizational support moderates between a knowledge-sharing culture and a high-performance organization.*

*H5a: Organizational support moderates between knowledge-sharing behavior and a high-performance organization.*

*H5b: Organizational support moderates between a knowledge-sharing strategy and a high-performance organization.*

*H5c: Organizational support moderates between mutual trust among members and a high-performance organization.*

## Methodology

### Measurements

After thoroughly evaluating prior studies, the questionnaire items concerning the self-directed team knowledge sharing culture, knowledge sharing strategy, mutual trust among members, sense of organizational support, and high-performance organization were selected. In accordance with prior research, all items were assessed on a seven-point Likert scale, with one indicating they strongly disagree and seven indicating they strongly agree. The content validity of the very first iteration of this instrument was pretested by 30 senior managers from companies that chose not to participate in the main research to ensure that it was valid. Respondents were asked to provide information on each question, including the structure, content, and text. Unclear items were rephrased in response to participant input. After revising the questionnaire in light of the pilot results, questionnaires were sent to 700 high-tech enterprises. The subsequent subsections go into further depth on each measurement.

Knowledge sharing culture use consists of six items adapted from Bartol and Wu (2009). At the same time, knowledge sharing strategy, knowledge sharing behavior, and mutual trust among members consist of 3, 3, and 3 items adapted from previous studies of Pinjani and Palvia (2013); Rehman et al. (2015), and Bhatti et al. (2020). Furthermore, the self-directed team consists of 10 items adapted from the study of Arnwine (1995). Finally, organizational support consists of 10 items adopted from Eisenberger et al. (1986), and high-performance organization consists of 8 items and adapted from the study of Guthrie et al. (2009). Table 1 contains the details of the variables included with the items studied in this research.

**TABLE 1:** Measurement constructs with items.

Constructs	Items	Source
Self-directed team	10	Arnwine (1995)
Knowledge sharing culture	6	Bartol and Wu (2009)
Knowledge sharing behavior	3	Bhatti et al. (2020)
Knowledge sharing strategy	3	Rehman et al. (2015)
Mutual trust among members	3	Pinjani and Palvia (2013)
Sense of organizational support	10	Eisenberger et al. (1986)
High-performance work (organization)	8	Guthrie et al. (2009)

### Data collection, sample size, and population of the study

This study gathered data from the senior management (marketing manager, HR manager, operational manager, finance manager) of high-tech enterprises publicly traded on the Shanghai Stock Exchange. Data were collected from employees of only 150 enterprises registered with the Shanghai Stock Exchange. Seven hundred fifty questionnaires were distributed in these enterprises, while 700 completed questionnaires were returned in two months. One hundred fifty of them had insufficient responses and were therefore not considered valid for further study. The total number of valid questionnaires received was 550, resulting in a response rate of 73 percent. There are numerous sample size ranges (Comrey and Lee, 2013). A sample size of less than 50 is regarded as weaker; 51–100 is considered poor, 101–200 is sufficient, 201–300 is good, 500 is very good, and 1,000 is considered excellent.

In order to determine the sample size in this research, G-power software was performed to identify the sample size because most scholars have recommended G-power for studies that applied using PLS-SEM (Hair et al., 2014; Henseler et al., 2015). Therefore, according to the G-Power analysis, a sample size of 550 would be sufficient to find a small effect size of 0.2 with an alpha (α err prob) of 0.154 and with a power (1-β err prob) of 0.82. As a result, the response rate of 550 is regarded as very good for the purpose of this research.

### Profile of respondents

61% of the participants were male, and 31% were female. Furthermore, 40% worked as human resources managers, 38% as finance managers, and 22% as marketing managers, among other positions. Regarding age, 6% were under the age of 25, 40% were between the ages of 31 and 40 years, 30% were between the ages of 41 and 50 years, and 24% were beyond the age of 50.53% had a bachelor’s degree, and 47% had a master’s degree. According to the survey results, around 11% of participants had five years or less of experience, 22% had between six and ten years of experience, 47% had between eleven and twenty years of experience, and 37% had more than twenty years of experience. The enterprises in concern were all from the private sector.

## Results

The stated hypotheses were statistically evaluated in this study with the help of SmartPLS 3.3.7. The rationale for choosing SmartPLS 3.3.7 is that it provides better results and is more capable of handling basic and large-scale procedures. Additionally, there is no necessity for a normality test (Risher and Hair, 2017; Wong, 2019). Furthermore, some previous studies have revealed that the PLS-SEM approach produces superior outcomes compared to the covariance-based technique (Hair et al., 2017; Rigdon et al., 2017). In PLS-SEM, two models are tested: measurement (outer) and structural (inner) models. In this study, we have employed both of these models.

### Measurement model (outer model)

Estimating a measurement (outer model) requires the measurement of three main validities: content validity, convergent validity, and third, discriminant validity.

#### Convergent validity

**FIGURE 2:**
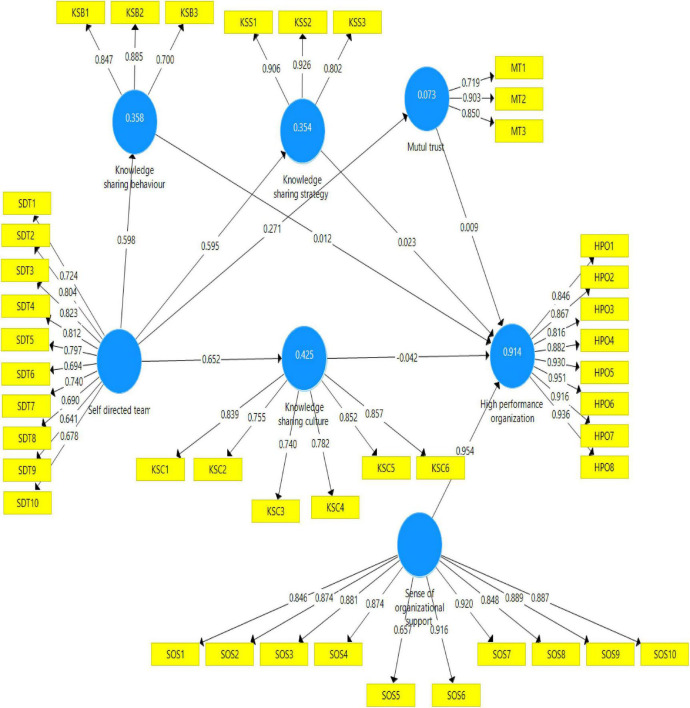
Measurement model.

In the context of convergent validity, a scenario exists where items of certain constructs effectively reflect their associated indicators (Hair et al., 2017). Convergence validity is required by three factors, according to Hair et al. (2020), including factor loadings, composite reliability (CR), and the average variance extracted (AVE). According to Hair et al. (2017), the AVE and factor loadings values must be more than 0.50, and the CR value must be greater than 0.70. For CR and AVE, any components with a factor loading of less than 0.50 must be removed in order to get a better result, as advised by the literature (Awang et al., 2015). Cronbach’s alpha value should be more than 0.60, according to Nunnally (1978). Figure 2 and Table 2 illustrate that this research meets the convergent validity requirement in terms of its findings.

**TABLE 2:** AVE, CR, and factor loadings.

Constructs	Items	Factor loadings	Cronbach alpha	CR	AVE
High performance organization	HPO1	0.846	0.964	0.969	0.799
	HPO2	0.867			
	HPO3	0.816			
	HPO4	0.882			
	HPO5	0.930			
	HPO6	0.951			
	HPO7	0.916			
	HPO8	0.936			
Know ledge sharing behavior	KSB1	0.847	0.739	0.854	0.663
	KSB2	0.885			
	KSB3	0.700			
Know ledge sharing culture	KSC1	0.839	0.891	0.917	0.649
	KSC2	0.755			
	KSC3	0.740			
	KSC4	0.782			
	KSC5	0.852			
	KSC6	0.857			
Know ledge sharing strategy	KSS1	0.906	0.853	0.911	0.774
	KSS2	0.926			
	KSS3	0.802			
Mutua l trust	MT1	0.719	0.776	0.866	0.685
	MT2	0.903			
	MT3	0.850			
Self- directed team	SDT1	0.724	0.910	0.924	0.552
	SDT2	0.804			
	SDT3	0.823			
	SDT4	0.812			
	SDT5	0.797			
	SDT6	0.694			
	SDT7	0.740			
	SDT8	0.690			
	SDT9	0.641			
	SDT10	0.678			
Sens of organizational support	SOS1	0.846	0.961	0.966	0.743
	SOS2	0.874			
	SOS3	0.881			
	SOS4	0.874			
	SOS5	0.657			
	SOS6	0.916			
	SOS7	0.920			
	SOS8	0.848			
	SOS9	0.889			
	SOS10	0.887			

#### Discriminant validity

A circumstance in which a researcher observes that every indicator of a theoretical framework (Figure 1) is significantly different is referred to as discriminant validity (Henseler et al., 2015). The term “discriminant validity” refers to the situation under which researchers determine whether or not two variables are statistically distinct. A variable’s real difference from those other variables is shown by how the variable differs from other variables based on quantitative measurements (Shaffer et al., 2016). Components of the specified variable should have a larger variance than the other variables in the theoretical model to be included. The present study assessed discriminant validity based on Fornell and Larcker’s (1981) recommendation. To apply this criterion, we must compare the diagonal higher values obtained by taking the square root of AVE with the values obtained underneath. The higher value of the diagonal must be bigger than the lower values of the same column and row in order to be valid. Table 3 reveals that the predefined requirements for discriminant validity are met in this investigation, as seen in the results (see Table 3).

**TABLE 3:** Discriminate validity (Fornell and Larcker, criteria).

Constructs	HPO	KSB	KSC	KSS	MT	SDT	SOS
HPO	*0.894*						
KSB	−0.108	*0.814*					
KSC	−0.142	0.747	*0.805*				
KSS	−0.088	0.671	0.633	*0.880*			
MT	0.015	0.260	0.223	0.283	*0.828*		
SDT	−0.103	0.598	0.652	0.595	0.271	*0.743*	
SOS	0.856	−0.112	−0.131	−0.099	0.005	−0.113	*0.862*

The use of italics indicates that the values in a certain column are higher. HPO, High-performance organization; KSB, Knowledge sharing behavior; KSC, Knowledge sharing culture; KSS, Knowledge sharing strategy; MT, mutual trust; STD, Self-directed team; SOS, Sense of organizational support.

Henseler et al. (2015) introduced a new approach for determining discriminant validity called the Heterotrait-Monotrait ratio, or HTMT. They verified that the standard measurement is inadequate for determining discriminant validity. Henseler et al. (2015) indicated that the standardized value for HTMT is 0.85 for conceptually distinct constructs and 0.90 for theoretically the same variables. Table 4 shows that all numbers are less than 0.85; thus, the discriminant validity criteria are contentious. Based on the findings of this research, it seems that the standardized requirements for the Heteroretrait-Monotrait ratio (HTMT) are met (see Table 4) (Hair et al., 2020).

**TABLE 4:** Discriminant validity Heterotrait-Monotrait ratio (HTMT) criteria.

Constructs	HPO	KSB	KSC	KSS	MT	SDT	SOS
HPO							
KSB	0.132						
KSC	0.157	0.827					
KSS	0.099	0.844	0.715				
MT	0.057	0.376	0.300	0.395			
SDT	0.110	0.696	0.675	0.643	0.299		
SOS	0.840	0.134	0.143	0.111	0.060	0.123	

HPO, High-performance organization; KSB, Knowledge sharing behavior; KSC, Knowledge sharing culture; KSS, Knowledge sharing strategy; MT, mutual trust; SDT, Self-directed team; SOS, Sense of organizational support.

### Structural model (inner model)

**FIGURE 3:**
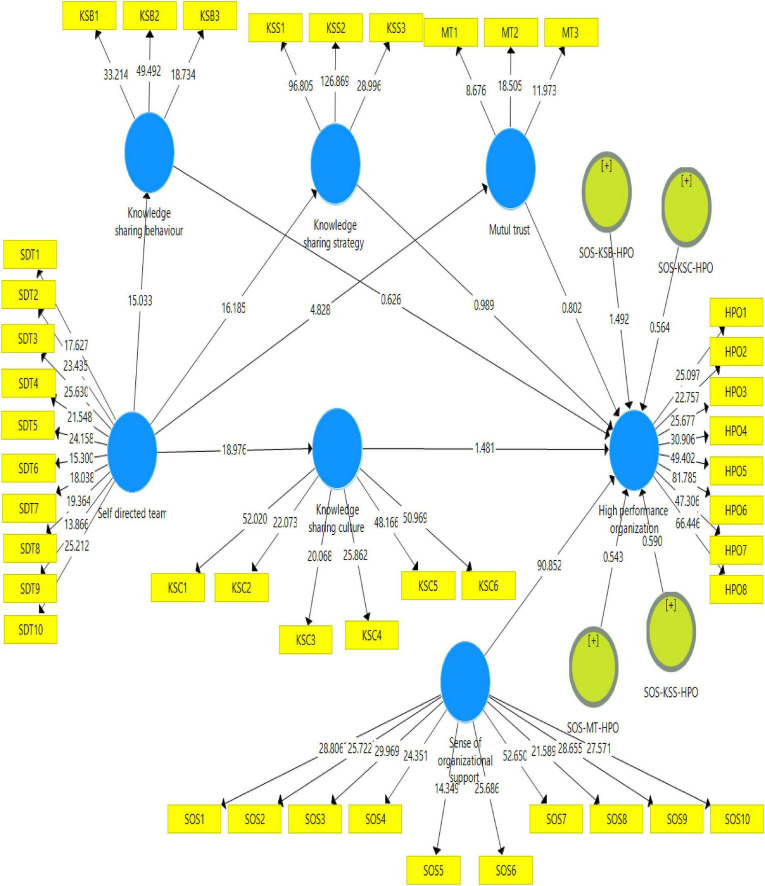
Structural model.

Following the execution of the measurement model in the previous part, we will now discuss the measures to verify the hypotheses. SmartPLS 3.3.7 is used to estimate the research model and assess the structural path, and the hypotheses are examined to do so. The measurement model (see Figure 3) was conducted in the previous part, and the structural model, also known as the inner model, is included in this portion. The researchers compute the p-value and the t-value in the inner model, that is used to test the hypotheses that have been suggested. Acceptance of the presented hypotheses is determined by the t-value being larger than 1.96, the p-value being less than 0.05, and vice versa, as shown in Table 5. Refer to Figure 3.

**TABLE 5:** Direct hypothesis relationships.

Hyp.	Paths	β-value	STDEV	t-value	P-value
H1	SDT -> HPO	0.001	0.001	0.012	0.101
H2	SDT -> KSC	0.652	0.034	18.976	0.000
H2a	SDT -> KSB	0.598	0.040	15.033	0.000
H2b	SDT -> KSS	0.595	0.037	16.185	0.000
H2c	SDT -> MT	0.271	0.056	4.828	0.000
H3	KSC -> HPO	−0.043	0.029	1.481	0.139
H3a	KSB -> HPO	0.017	0.027	0.626	0.531
H3b	KSS -> HPO	0.026	0.026	0.989	0.323
H3c	MT -> HPO	0.013	0.017	0.802	0.423

This study has nine direct hypotheses, four indirect (mediating) hypotheses, and four moderating hypotheses and such complexity of the model. Figure 3 and Table 5 demonstrate the t-values and β-values to confirm whether the hypotheses are supported or not. To estimate the theoretical framework and analyze the structural path, the hypotheses are tested using SmartPLS 3.3.7. Following the measurement model, this section covers the structural model, computing t-value and p-value to test the hypotheses. The relationship is considered significant if the p-value is less than 0.05 or the t-value more than 1.96. SDT has an insignificant influence on high performance organization (β-values 0.001, *p* < 0.101, and t-value 0.012), H1 is rejected, While, STD on KSC, KSB, KSS and MT positive and significant relationship, (β-values 0.652, *p* < 0.000, and t-value 18.976), and KSB (β-values 0.598, *p* < 0.000, and t-value 15.033), KSS (β-values 0.595, *p* < 0.000, and t-value 16.185), MT (β-values 0.271, *p* < 0.000, and t-value 4.828), H2, H2a, H2b and H2c is accepted.

PLS-SEM bootstrapping was used to perform the mediation analysis. The fourth hypothesis examined the knowledge-sharing culture’s mediating effect and its dimensions. Bootstrapping makes testing mediation hypotheses easier (Picón et al., 2014). The mediators’ 95% confidence intervals (percentiles) are calculated using this approach. A summary of hypotheses evaluated via mediating effect of a knowledge-sharing culture can be seen in Table 6. In order to validate mediation between the constructs, there are two processes involved. It is essential to determine the significance of the direct effect in the first step, and then it is essential to define the significance of the indirect effect in the second step, and the strength of the mediating construct will be evaluated in the final step. In the end, mediation has taken place if it is determined that there was a significant direct effect in addition to a significant indirect effect. Unlikely, if the indirect effect is not significant, it suggests that no mediation happens. It is also important to realize that mediation is present if the signs of the confidence intervals (LCI and UPCI) are the same. This means that both LCI and UPCI must be either positive or negative. If, on the other hand, one sign is positive and the other is negative, this indicates no mediation (Picón et al., 2014). As in Table 6, we can see the negative sign of LCI and positive signs of UCI, which means that there is no mediation of knowledge-sharing culture with its dimensions.

**TABLE 6:** Mediation hypothesis relationships.

Hyp.	Paths	β-value	STDEV	T-value	*P*-value	LCI 2.50%	UCI 97.50%
H4	SDT -> KSC -> HPO	−0.028	0.019	1.459	0.145	−0.066	0.005
H4a	SDT -> KSB -> HPO	0.010	0.016	0.619	0.536	−0.021	0.043
H4b	SDT -> KSS -> HPO	0.015	0.016	0.974	0.331	−0.016	0.049
H4c	SDT -> MT -> HPO	0.004	0.005	0.768	0.443	−0.005	0.014

**FIGURE 4:**
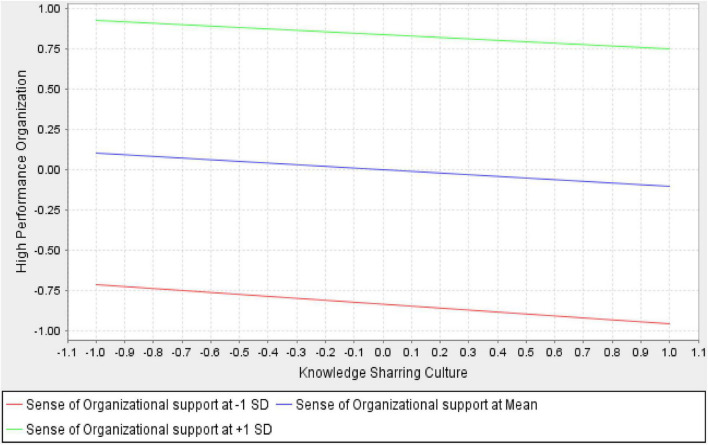
Sense of organizational support moderates between a knowledge-sharing culture and a high-performance organization.

Sense of organizational support (SOS) moderates the relationship between KSC and high-performance organizations (β-values 0.019, *p* < 0.573, and t-value 0.564), not supporting H2 Figure 4. Table 7 demonstrates that KSB, KSS and MT has the negative and insignificant relation with high performance organization, (β-values −0.032, *p* < 0.136, and t-value 1.492, β-values −0.017, *p* < 0.555, and t-value 0.590, β-values −0.009, *p* < 0.587, and t-value 0.543). Moreover, the result of H5, H5a, H5b, and in Table 7 reveals that KSC, KSB, KSS, and MT does not moderate the relationship between SOS and HPO. Therefore, H5 with its sub-hypothesis is rejected.

**TABLE 7:** Structural equation model results for moderation.

Hyp.	Paths	β-value	STDEV	T-value	*P*-value
H5	SOS*KSC -> HPO	0.019	0.033	0.564	0.573
H5a	SOS*KSB -> HPO	−0.032	0.021	1.492	0.136
H5b	SOS*KSS -> HPO	−0.017	0.029	0.590	0.555
H5c	SOS*MT -> HPO	−0.009	0.016	0.543	0.587

#### The predictive relevance and effect size

In the present study, the researcher focuses on two situations to obtain the predictive relevance of theoretical models of studies, namely cross-validated redundancy and R-squared correlation coefficients. The variance that describes all independent constructs is referred to as the R-square. Table 6 demonstrates that (91 percent, 36 percent, 42 percent, and 35 percent) of the variance is explained separately by each independent variable. Cohen (1988) defines R-square as follows: a value of R^2^ between 0.02 and 0.13 is regarded weak, 0.13–0.26 is considered moderate, and more than 0.26 is considered substantial. In this research, the R^2^ values of all variables, except for mutual trust, are substantial.

Furthermore, the cross-validated redundancy of the research model is evaluated in order to estimate the overall accuracy of the model. Cross-validated redundancy is determined in SmartPLS using the blindfolding approach. This procedure necessitates the removal of a small number of data values that are considered missing values on the researcher’s end. Q^2^ value must be larger than zero to be valid (Henseler et al., 2015). As shown in Table 8, the present research satisfies the predefined evaluation criteria.

**TABLE 8:** R^2^ and Q^2^.

Constructs	R-Square	R-Square adjusted	Q^2^
High-performance organization	0.914	0.913	0.724
Knowledge sharing behavior	0.358	0.355	0.228
Knowledge sharing culture	0.425	0.423	0.271
Knowledge sharing strategy	0.354	0.352	0.269
Mutual trust	0.073	0.070	0.046

## Discussion and conclusion

This study aims to determine the direct impact of self-directed teams and a culture of knowledge sharing on high-performance organizations. Additionally, investigate the mediating effect of knowledge-sharing culture on the relationship between self-directed teams and high-performance organizations. Moreover, examine the moderating effect of a sense of organizational support on the relationship between knowledge-sharing culture and high-performance organization in the context of Chinese firms. The focus of the present investigation is quantitative and descriptive. As a result, only four hypotheses, H2, H2a, H2b, and H2c, are acceptable, while all other hypotheses are rejected.

The primary objective of this study was to explore the relationship between a self-directed, knowledge-sharing culture with high-performance organizations. According to this research findings, self-directed teams positively and significantly affect knowledge sharing culture, knowledge sharing behavior, knowledge sharing strategy, and mutual trust. However, while we confirm with this study, knowledge-sharing culture, in all its dimensions, does not mediate the relationship between a self-directed team and a high-performance organization. Furthermore, we employed a sense of organizational support as a moderator for the relationship between knowledge-sharing culture and high-performance organizations. At the same time, we confirm on behalf of this study sense of organizational support is not moderated by the relationship between knowledge-sharing culture and high-performance organizations.

### Theoretical and managerial implications

The current study’s findings have a wide range of practical implications for high-tech businesses. This research guides high-tech enterprises worldwide that want to improve their long-term sales by considering self-directed teams, a culture of knowledge sharing, a sense of organizational support, and a high-performance organization. According to Buenechea-Elberdin et al. (2018), considering knowledge-sharing culture only is insufficient to accurately predict high-performance organizations. Because many other factors play an important role in determining the performance of high-tech enterprises, this is the reason why we employed the sense of organizational support as moderating factor in this study. According to the findings of this study, a high-tech enterprise should create a sense of organizational support to improve its performance of the enterprises. This shows that the sense of organizational support positively affects the performance of high-tech enterprises. The current study’s findings are beneficial for high-tech enterprises because they show that self-directed team management interventions should be easy to understand for organizations trying to improve high-performance organizations through greater collaboration. The findings of this study are relevant to Chinese enterprises and other economies worldwide because this framework is widely recognized throughout the research, and concerns about self-directed teams and the sense of organizational support exist in every sector emerging worldwide.

### Limitations future suggestions

As is the case with all research, the present study also has some limitations. First and foremost, we assessed all indicators through a questionnaire. Although this strategy will likely change the general methodology, prior research supported our decision to adopt this approach. According to Manstead (2018), survey questionnaire data is useful for researching psychological states’ dimensions since it describes individuals’ subjective states that influence their behavior. Nevertheless, we took all necessary precautions to rule out the possibility of common method variance. Another potential limitation of the study is that it focused on quantitative research and used a non-probability research design, which is considered a limitation of this study. This study is similar to other earlier studies in that it has several limitations that should be taken into consideration in future research using these components. The current study investigated the direct impact of self-directed teams, knowledge-sharing cultures, and a sense of organizational support on high-performance organizations (work). In the future, it would be necessary to include other mediating or moderating factors and theories between these relationships to understand them fully. Future research might circumvent this constraint by assessing actual knowledge-sharing behavior rather than the view of knowledge-sharing culture as a mediator exclusively, as is currently the case. Subjective measurements are extensively utilized in the literature; in the lack of objective data, self-reported measures may serve as an adequate substitute and can be just as accurate as the objective measures employed in the study. Furthermore, future studies might use various data strategies, such as structured interviews, to limit the likelihood of incorrect interpretations of the research constructs and explicitly explain the distinctions between them. Moreover, future research might include other aspects, such as job satisfaction and employee commitment, to construct a more appropriate framework.

## Data availability statement

The original contributions presented in this study are included in the article/supplementary material, further inquiries can be directed to the corresponding author.

## Author contributions

BM wrote the manuscript, collected the data, and performed the analysis. ZJ conceived the draft and contributed to supervision. CY contributed to the methodology design. YY contributed to the data. YA contributed to the discussion. All authors contributed to the article and approved the submitted version.
